# Robust and Highly Stretchable Chitosan Nanofiber/Alumina-Coated Silica/Carboxylated Poly (Vinyl Alcohol)/Borax Composite Hydrogels Constructed by Multiple Crosslinking

**DOI:** 10.3390/gels8010006

**Published:** 2021-12-22

**Authors:** Hiroyuki Takeno, Nagisa Suto

**Affiliations:** 1 Division of Molecular Science, Graduate School of Science and Technology, Gunma University, Kiryu 376-8515, Japan; t191a047@gunma-u.ac.jp; 2 Center for Food Science and Wellness, Gunma University, 4-2 Aramaki, Maebashi 371-8510, Japan

**Keywords:** chitosan nanofiber, composite hydrogel, nanoparticles, tough hydrogels, multiple crosslinking

## Abstract

We investigated the mechanical and structural properties of composite hydrogels composed of chitosan nanofiber (ChsNF), positively charged alumina-coated silica (ac-SiO_2_) nanoparticles, carboxylated poly (vinyl alcohol) (cPVA), and borax. ChsNF/cPVA/borax hydrogels without ac-SiO_2_ exhibited high Young’s modulus but poor elongation, whereas cPVA/ac-SiO_2_/borax hydrogels without ChsNF had moderate Young’s modulus but high elongation. ChsNF/ac-SiO_2_/cPVA/borax hydrogels using both ChsNF and ac-SiO_2_ as reinforcement agents exhibited high extensibility (930%) and high Young′s modulus beyond 1 MPa at a high ac-SiO_2_ concentration. The network was formed by multiple crosslinking such as the complexation between borate and cPVA, the ionic complexation between ac-SiO_2_ and cPVA, and the hydrogen bond between ChsNF and cPVA. Structural analysis by synchrotron small-angle X-ray scattering revealed that the nanostructural inhomogeneity in ChsNF/ac-SiO_2_/cPVA/borax hydrogel was suppressed compared to those of the ChsNF/cPVA/borax and cPVA/ac-SiO_2_/borax hydrogels.

## 1. Introduction

Hydrogels composed of biocompatible polymers have attracted numerous researchers and engineers because of potential applications in the biomedical field. Chitosan (Chs), which is known as one of the biocompatible polymers, is produced by alkali deacetylation of chitin, the major component in the exoskeleton of crustaceans [1]. Chs is soluble in acidic aqueous solutions, where it has positive charges due to the protonation of amine groups on the backbone. Accordingly, Chs can form a polyelectrolyte complex with the oppositely (negatively) charged polyelectrolyte such as *κ*-carrageenan or xanthan gum [2,3]. However, generally, the mechanical strength of polyelectrolyte complex gels is comparatively weak. As one of the methods to enhance the mechanical strength of polymer hydrogels or produce mechanically tough polymer hydrogels, the addition of reinforcing agents such as inorganic nanoparticles [4,5] or clay nanoparticles [6,7,8] is effective. To acquire effective reinforcement effects, it is necessary to disperse the nanoparticles and to connect between the nanoparticles and the polymer [9,10]. In these composite hydrogels, the nanoparticles act as a multiple-crosslinker, i.e., a lot of polymer chains are attached to one nanoparticle, i.e., multiple crosslinking points are formed, so that mechanically tough hydrogels are formed. Additionally, our previous studies clarified that the molecular mass of the constituent polymer is a key factor in the enhancement of mechanical performance [11,12]. Besides, we recently reported that composite hydrogels using two kinds of reinforcing agents such as clay and silica nanoparticles were robust and highly stretchable [13,14]; two identically charged nanoparticles formed multiple crosslinking and suppressed inhomogeneities in the composite hydrogels [13,14]. Thus, the construction of the gel network by multiple crosslinking was effective in fabricating tough polymer hydrogels.

In the case of biocompatible polymer hydrogels, nanofibers have been used to enhance the mechanical performance, e.g., mechanically tough composite hydrogels using cellulose nanofiber have been developed [9,15,16]. Similarly, nanofibers of chitosan can reinforce polymer hydrogels [17,18]. Nitta et al. used a chitosan nanofiber (ChsNF) to reinforce the mechanical strength of polyethylene glycol (PEG) hydrogels so that the compressive modulus and fracture stress of the ChsNF/PEG composite hydrogels attained the values of ~10 kPa and ~15 kPa, respectively [17]. Zhou and Wu showed that the compressive stress of a ChsNF/poly (acrylamide) (PAM) composite hydrogel at 95% strain attained the value of ~50 kPa, which was 7.7 times higher than that of PAM hydrogel without ChsNF [18].

In this study, we tried to enhance the mechanical performance of the hydrogels composed of chitosan and carboxylated poly (vinyl alcohol) (cPVA). For this purpose, we used ChsNF and positively charged alumina-coated silica nanoparticles (ac-SiO_2_) as reinforcing agents. As a result, we found that the ChsNF/ac-SiO_2_/cPVA/borax composite hydrogels were highly stretchable and robust.

## 2. Results and Discussion

### 2.1. Mechanical Properties of the Composite Hydrogels

**Figure 1:**
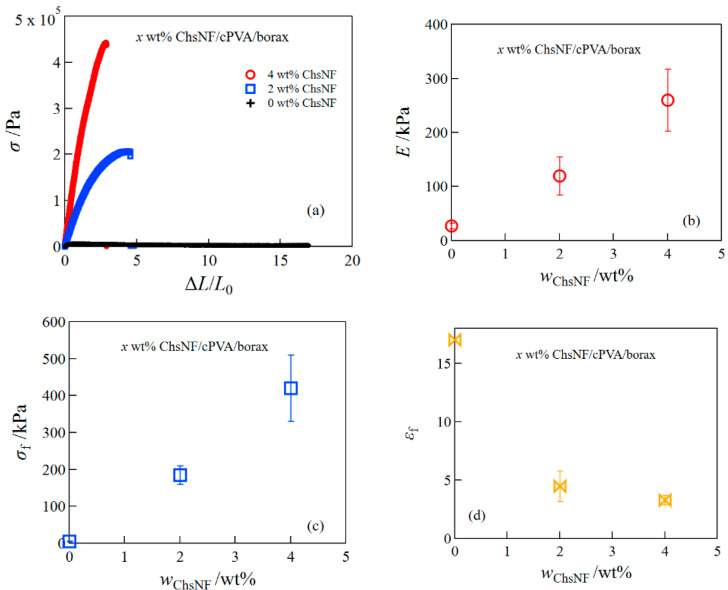
Tensile stress–strain curves (**a**), the Young′s modulus (**b**), fracture stress (**c**), and fracture strain (**d**) for ChsNF/cPVA/borax hydrogels.

First, we examined the effect of the addition of ChsNF on the mechanical properties of cPVA/borax hydrogels. Figure 1 depicts representative tensile stress–strain curves (a), the Young′s modulus *E* (b), the fracture stress *σ*_f_ (c), and the fracture strain *ε*_f_ (d) for ChsNF/cPVA/borax hydrogels at different ChsNF concentrations. Both *E* and *σ*_f_ largely increased with increasing ChsNF concentrations but *ε*_f_ dramatically decreased. This behavior has often been seen for many composite hydrogels; although the increase in the content of the reinforcing agent leads to enhancement of the mechanical strength, the degree of elongation lowers because of the difficulty in the dispersion of the reinforcing agent.

**Figure 2:**
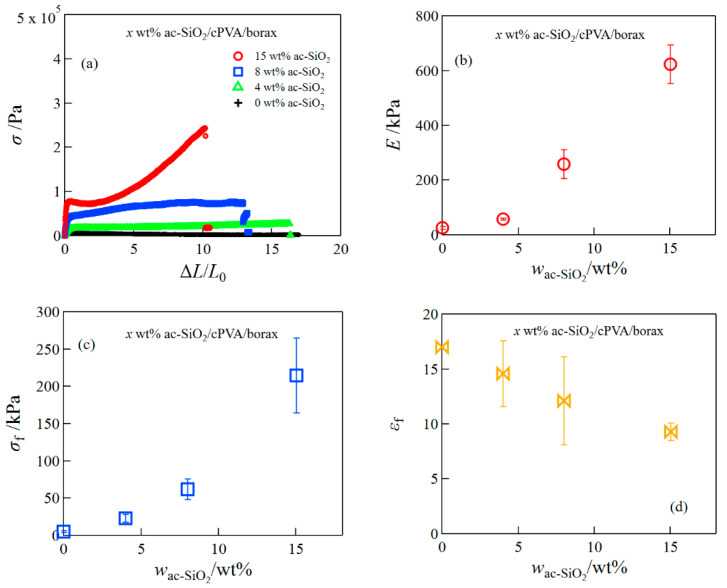
Tensile stress–strain curves (**a**), the Young′s modulus (**b**), fracture stress (**c**), and fracture strain (**d**) for ac-SiO_2_/cPVA/borax hydrogels.

Next, we investigated the effect of the addition of ac-SiO_2_ nanoparticles on the mechanical properties of cPVA/borax hydrogels (Figure 2). Similarly, both *E* and *σ*_f_, for the ac-SiO_2_/cPVA/borax hydrogels, significantly increased with the increase in ac-SiO_2_. The increase in *E* was remarkably larger at higher ac-SiO_2_ concentrations. At high concentrations of nanoparticles, the inter-particle distance became closer, so that many polymer chains could be attached to one nanoparticle, i.e., the number of crosslinking points increased. *ε*_f_ showed a gradual decrease with the increase in the ac-SiO_2_ concentration; the degree of elongation exhibited a value of ~1000% even at high concentrations of ac-SiO_2_. Although the tensile stress of ac-SiO_2_/cPVA/borax hydrogels increased with the addition of ac-SiO_2_, the reinforcement effect was lower compared to ChsNF/cPVA/borax hydrogels.

**Figure 3:**
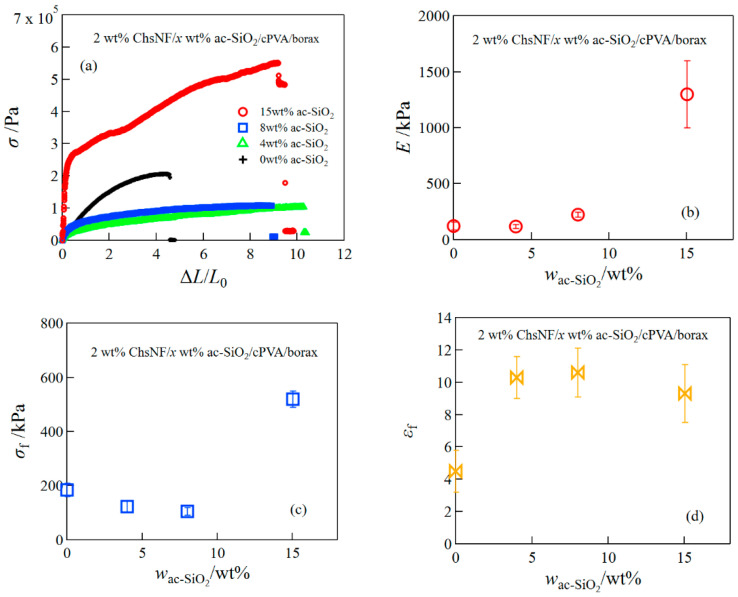
Tensile stress–strain curves (**a**), the Young′s modulus (**b**), fracture stress (**c**), and fracture strain (**d**) for ChsNF/ac-SiO_2_/cPVA/borax hydrogels.

For further improvement of the mechanical performance, i.e., expecting the composite hydrogels with both high mechanical strength and high extensibility, we prepared ChsNF/ac-SiO_2_/cPVA/borax hydrogels using both ChsNF and ac-SiO_2_ nanoparticles as reinforcing agents. Figure 3 depicts the representative stress–strain curves (a), the Young′s modulus (b), the fracture stress (c), and the fracture strain for 2 wt% ChsNF/ac-SiO_2_/cPVA/borax hydrogels at different ac-SiO_2_ concentrations. The ChsNF/ac-SiO_2_/cPVA/borax hydrogels exhibited excellent mechanical properties with high mechanical strength and high elongation. Especially, the composite hydrogel at 15 wt% ac-SiO_2_ obtained the Young’s modulus of 1.3 MPa and an elongation of 930%.

### 2.2. Fourier-Transform Infrared (FT-IR) Spectroscopy

**Figure 4:**
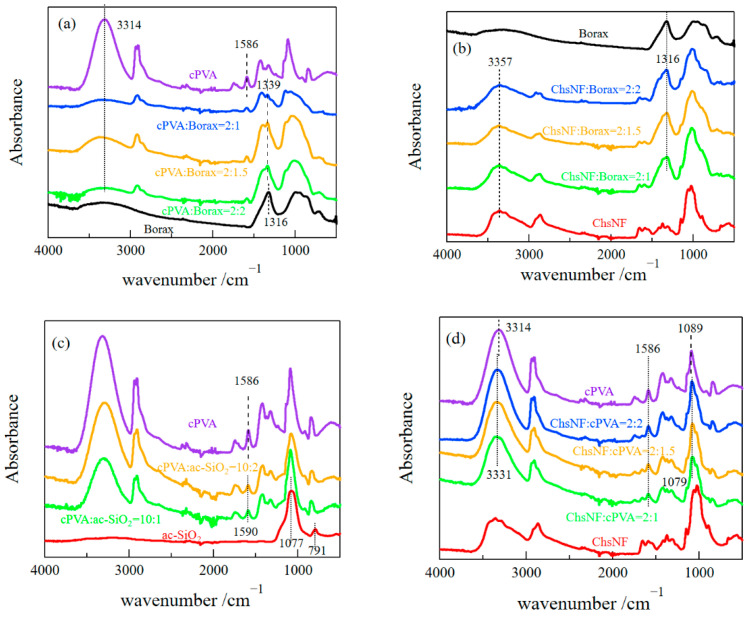
FT-IR spectra for cPVA/borax (**a**), ChsNF/borax (**b**), cPVA/ac-SiO_2_ (**c**), and ChsNF/cPVA (**d**).

We performed FT-IR measurements to examine the interactions between different components. Figure 4a,b depict the FT-IR spectra for the cPVA/borax and ChsNF/borax systems. A characteristic peak was observed at 1339 cm^−1^ for the cPVA/borax systems; the intensity became larger as the borax content increased. This band was assigned to the asymmetric stretching vibration of B-O-C, indicating the tetrahedral complexation between PVA and borate [19,20,21]. For the ChsNF/borax system, the characteristic band was observed at 1316 cm^−1^, which was at the same position as for pure borax. This result suggested that tetrahedral complexation was, to a significant extent, not formed between ChsNF and borate. Figure 4c,d show the FT-IR spectra for the cPVA/ac-SiO_2_ and ChsNF/cPVA systems. The characteristic bands observed at 1077 and 791 cm^−1^ for pure ac-SiO_2_ were ascribed to antisymmetric and symmetric Si-O-Si (or Si-O-Al) stretching vibrations [13,22,23,24]. The characteristic band at 3314 cm^−1^ for pure cPVA was assigned to the stretching vibration of hydrogen-bonded hydroxyl groups, whereas the band at 1586 cm^−1^ was assigned to the COO^–^ antisymmetric stretching vibration [25]. The characteristic band of the COO^–^ antisymmetric stretching vibration was shifted to a higher wavenumber (1590 cm^−1^) for the cPVA/ac-SiO_2_ system, whereas it was observed at the same wavenumber for the ChsNF/cPVA system. These results suggested that ion complexation between cPVA and ac-SiO_2_ was formed, whereas it was not formed between cPVA and ChsNF. The band at 3314 cm^−1^ arising from the OH stretching vibration for pure cPVA was shifted to a higher wavenumber (3331 cm^−1^) for ChsNF/cPVA. Besides, the band observed at 1089 cm^−1^ for pure cPVA, which was assigned to the stretching vibration of C-O [26,27], was shifted to a lower value (1079 cm^−1^) for ChsNF/cPVA. This result suggested hydrogen bonding between ChsNF and cPVA.

### 2.3. Synchrotron Small-Angle X-ray Scattering and Wide-Angle X-ray Scattering

**Figure 5:**
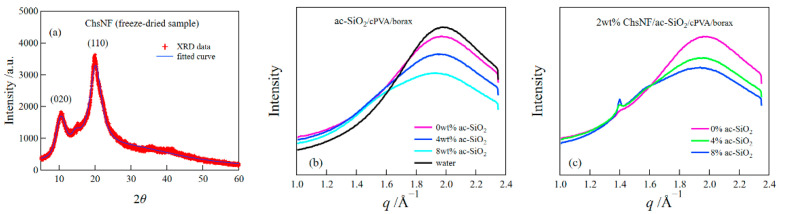
XRD curve for ChsNF powder (**a**), WAXS curves for ac-SiO_2_/cPVA/borax hydrogels (**b**), and ChsNF/ac-SiO_2_/cPVA/borax (**c**) hydrogels.

Synchrotron SAXS and WAXS measurements were performed to examine the structures of the composite hydrogels. Firstly, we conducted the XRD measurement for a freeze-dried sample of ChsNF dispersion (Figure 5a). The XRD curve had sharp diffraction peaks at 2*θ* ≈ 10.6° and 20.0°, which were assigned to the (020) and (110) reflections, respectively [28,29]. We estimated the crystallinity of ChsNF from the XRD curve and obtained a value of 35%.

Figure 5b depicts the WAXS curves for the ac-SiO_2_/cPVA/borax hydrogels at different ac-SiO_2_ concentrations. For comparison, the WAXS curve for water is appended in the figure. The figure does not show any peak except for the amorphous peak of water, suggesting that cPVA and ac-SiO_2_ were amorphous in the hydrogels. Figure 5c shows the WAXS curves for the ChsNF/ac-SiO_2_/cPVA/borax hydrogels at different ac-SiO_2_ concentrations. All the curves were found to have a small peak at *q* = 1.4 Å^−1^, which corresponded to the (110) reflection of the chitosan crystal. The peak intensity slightly increased with the increase in the ac-SiO_2_ concentrations. This result suggests that the addition of ac-SiO_2_ seemingly induced the crystallization of ChsNF. However, the situation may have been unexpected; the pH values only showed a slight decrease with the addition of ac-SiO_2_ (pH = 8.5 for 0 wt% ac-SiO_2_ and 8.2 for 8 wt% ac-SiO_2_). As another possibility, a preferential orientation of ChsNF may have influenced the intensity of the Bragg reflection, although we could not estimate the orientation from the 2D SAXS images of the ChsNF/ac-SiO_2_/cPVA/borax hydrogels; this was because the X-ray pattern of the composite hydrogels was dominated by the scattering intensity of ac-SiO_2_, as mentioned below.

**Figure 6:**
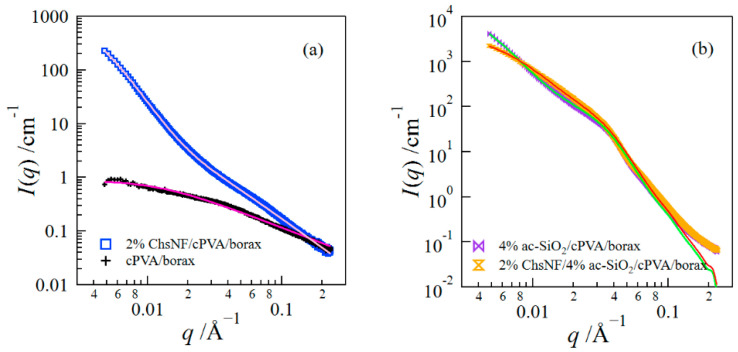
SAXS curves for cPVA/borax and ChSNF/cPVA/borax hydrogels (**a**), and for ac-SiO_2_/cPVA/borax hydrogels and ChsNF/ac-SiO_2_/cPVA/borax hydrogels (**b**).

Figure 6a depicts the SAXS profiles of the cPVA/borax and ChsNF/cPVA/borax hydrogels. First, the analysis of the SAXS data for cPVA/borax hydrogel was conducted. Shibayama et al. analyzed the small-angle neutron scattering data for PVA/borax hydrogels using a generalized Zimm model for fractals with the fractal dimension of *D* [30]. According to the model, the scattering function is described as follows:

(1) $$I\left(q\right)=\frac{I\left(0\right)}{{\left\{1+\frac{\left(D+1\right)}{3}\xi^{2}q^{2}\right\}}^{D/2}}$$

where *I*(0) and *q* are the scattering intensity at *q* = 0 and the magnitude of the wavevector defined by *q* = 4π sin (*θ*/2)/*λ*. Here, *θ* and *λ* are the scattering angle and the wavelength, respectively. *ξ* is the correlation length, which represents the spatial length of concentration fluctuations. We carried out the fitting analysis for the SAXS data of cPVA/borax hydrogel using Equation (1); the fitted curve represented the scattering data well. As a result, the values of *D* = 1.0 and *ξ* = 101 Å were obtained. The latter value was almost the same as that of the PVA/borax gel [30]. The SAXS intensity for the ChsNF/cPVA/borax hydrogel largely increased compared to that of the cPVA/borax hydrogel; in particular, the scattering intensity at small *q* increased upward, reflecting the inhomogeneous distribution of ChsNF in the gel. Accordingly, we analyzed the SAXS data with the Debye–Buche function that had been used for the analysis of the inhomogeneous structure [31]

(2) $$I_{DB}\left(q\right)=\frac{I_{1}}{{\left(1+{\xi_{DB}}^{2}q^{2}\right)}^{2}}$$

where *I*_1_ is the prefactor of the scattering intensity and *ξ_DB_*—denotes a parameter that characterizes the spatial length of the inhomogeneous structure in the model. The fitting analysis using Equations (1) and (2) was conducted for the SAXS curve of the ChsNF/cPVA/borax hydrogel, so that we obtained the values of *D* = 1.8, *ξ* = 47 Å and *ξ_DB_* = 275 Å. Thus, the SAXS analysis revealed that the inhomogeneous structure, with a size of tens of nanometers, was formed in the composite gel, which have may have caused poor elongation for this composite hydrogel; the lowering of elongation due to an inhomogeneous distribution of reinforcing agents was also reported in previous studies [12,32]. The correlation length of Equation (1) for the ChsNF/cPVA/borax hydrogel was smaller than that of the cPVA/borax hydrogel. The hydrogen bond between ChsNF and cPVA shown by the FT-IR measurements may have suppressed the concentration fluctuations in the gel network. Figure 6b shows the scattering curves for the ac-SiO_2_/cPVA/borax and ChsNF/ac-SiO_2_/cPVA/borax hydrogels. The scattering profiles for both hydrogels were similar except for the scattering behavior at small *q.* This result suggests that their scattering curves were significantly dominated by the scattering from ac-SiO_2_ nanoparticles constituted of heavy atoms; heavier atoms have a larger scattering length in X-ray scattering [33].

The scattering function of spherical particles with a radius of *R* can be expressed by

(3) $$I\left(q\right)=I_{1}P_{sphere}\left(q\right)S\left(q\right)$$

with

(4) $$P_{sphere}\left(q\right)={\left[\frac{3\left\{\sin\left(qR\right)-qR\cos\left(qR\right)\;\right\}}{{\left(qR\right)}^{3}}\right]}^{2}$$

where *I*_1_ is a prefactor of the scattering intensity. *P_sphere_*(*q*) and *S*(*q*) are the form factor and the structure factor of spherical particles; the former and the latter correspond to the scattering from the intra-particle interference and the inter-particle interference, respectively. We considered the size distribution of the spherical particles using a Gaussian distribution with the mean radius of *R*_0_ and the standard deviation *σ*. Furthermore, we adopted the Percus–Yevick (PY) hard-sphere model [34] to calculate the structure factor *S*(*q*). The detailed representation of the function was described elsewhere [13,14]; in short, the function can be expressed using parameters of an interaction radius *R*_HS_ and the volume fraction of spheres *ϕ*.

The scattering curve for the ac-SiO_2_/cPVA/borax hydrogel was analyzed with a combination of the PY and DB models, i.e., Equations (2)–(4). The fitted curve is shown in Figure 6b, and the obtained parameters are summarized in Table 1. The mean radius of the ac-SiO_2_ nanoparticles obtained in the fitting analysis agreed well with the value (12 nm) of the particle diameter shown in the product catalog. Subsequently, we analyzed the scattering curve for the ChsNF/ac-SiO_2_/cPVA/borax hydrogel using Equations (2)–(4). In the analysis, we fixed the parameters in Equations (3) and (4) using the values obtained in the analysis of the ac-SiO_2_/cPVA/borax hydrogel, because the scattering curves for both composite hydrogels were almost the same except for the scattering behavior at small *q* arising from the inhomogeneous structure that could be expressed by the DB model. Consequently, the spatial length of the inhomogeneous structure obtained in the fitting analysis for the ChsNF/ac-SiO_2_/cPVA/borax hydrogel was much smaller, which suggested that the addition of ChsNF to the ac-SiO_2_/cPVA/borax hydrogel suppressed the inhomogeneity in the composite hydrogel. Thus, the ChsNF/ac-SiO_2_/cPVA/borax hydrogel possessed excellent mechanical performance, having both robustness and a high degree of elongation, as a result of the lowering of the inhomogeneity in the gel.

## 3. Summary

We investigated the mechanical and structural properties of composite hydrogels using chitosan nanofibers and alumina–coated silica nanoparticles as reinforcing agents and borax as a crosslinker. The composite hydrogels exhibited mechanically robust and highly stretchable properties. This study showed that the combined use of bio-nanofiber and inorganic nanoparticles as reinforcing agents is effective in the fabrication of robust and highly stretchable composite hydrogels. The composite hydrogels were constructed by the multiple crosslinking composed of ion complexation between cPVA and ac-SiO_2_, the hydrogen bond between ChsNF and cPVA, and the complexation between cPVA and borate. Synchrotron SAXS analysis revealed that the inhomogeneity in the ChsNF/ac-SiO_2_/cPVA/borax hydrogel was significantly suppressed so that the composite hydrogel exhibited excellent mechanical performance with both high mechanical strength and high degrees of elongation.

## 4. Experimental

### 4.1. Materials

In this study, we used chitosan nanofiber with a diameter of 20–50 nm (ChsNF) purchased from Sugino Machine Ltd. Carboxylated poly (vinyl alcohol) (cPVA) with a saponification value larger than 99% (Gohsenol T-330H) and alumina-coated silica nanoparticles (ac-SiO_2_) with particle sizes of 12 nm (SNOWTEX ST-AK) were kindly supplied from Mitsubishi Chemical Corp. and Nissan Chemical Corp., respectively. Sodium tetraborate decahydrate (borax) was purchased from Kanto Chemical Co., Inc. (Tokyo, Japan).

### 4.2. Gel Preparation

After a ChsNF suspension and an ac-SiO_2_ suspension were added into a vial, ChsNF and ac-SiO_2_ nanoparticles were dispersed using an ultrasonic homogenizer (QSONICA Model Q55) for 30 min. Afterward, cPVA was added to the ChsNF/ac-SiO_2_ suspension and dissolved at 90 °C. After the suspension was condensed in a vacuum oven to reach the desired concentration, borax was added. After the mixture was thoroughly mixed using a glass rod, it was placed in a mold with 1 mm thickness and was pressed at 70 °C. The final concentrations of cPVA and borax were 10 wt% and 3 wt%, respectively. The concentrations of ChsNF and ac-SiO_2_ are shown in the text. The pH values for the ChsNF/ac-SiO_2_/cPVA/borax hydrogels were measured with a PH mater (F-71, Horiba, Kyoto, Japan) and a pH electrode (ISFET 0040-10D, Horiba).

### 4.3. Tensile Tests

We performed tensile tests for the composite hydrogels using TENSILE TESTER STM-20 (ORIENTEC). The measurements were conducted at a stretching speed of 10 mm/min for the specimens with 1 mm thickness, 10 mm length, and 15 mm width. The tensile stress *σ* and strain *ε* were calculated from the relations of *σ* = *F*/*S*_0_ and *ε* = ∆*L*/*L*_0_, where *F* and ∆*L* are the tensile force and the deformation, respectively. *S*_0_ and *L*_0_ denote the initial area and initial length of the test specimen, respectively. The Young′s modulus *E* was estimated from the slope of the stress–strain curve at small strains. The average values of *E*, *σ*_f_, and *ε*_f_ were obtained from three tests.

### 4.4. Fourier-Transform Infrared (FT-IR) Measurements

FT-IR spectroscopy (JASCO FT/IR 4700) was used to examine the interactions between different components using the attenuated total reflection (ATR) method. The FT-IR spectra were recorded in the wavenumber range of 500–4000 cm^−1^. Freeze-dried samples were used for FT-IR measurements.

### 4.5. X-ray Diffraction Measurements

X-ray diffraction (XRD) measurement was conducted to explore the structure of ChsNF using an X-ray diffractometer (RIGAKU, RINT2200VF) at the Center for Instrumental Analysis of Gunma University. CuK α radiation was used in this measurement, and the diffracted intensity was detected at the diffraction angles of 5°–60°. The sample for XRD measurement was prepared as follows: a ChsNF suspension was freeze-dried, and the freeze-dried sample was filled in an aluminum spacer for XRD measurements. The crystallinity of ChsNF was estimated from the ratio of the area of crystalline peaks to the whole area.

### 4.6. Synchrotron Small-Angle X-ray Scattering/Wide-Angle X-ray Scattering

Synchrotron SAXS and WAXS measurements were performed to investigate the structure of the composite hydrogels. The experiments were conducted at the beamline 6A at the photon factory of the High Energy Accelerator Research Organization (KEK) in Tsukuba, Japan. An X-ray beam with a wavelength of 1.5 Å was used for the measurements, and the scattered intensity was detected using two-dimensional detectors—PILATUS 1M for SAXS and PILATUS 100K for WAXS. The detected X-ray images were circularly averaged to obtain the scattering curves as a function of *q* [35]. Moreover, the scattering intensity was corrected by the beam intensity, transmittance, and background scattering, and was reduced to the absolute units [36].

## Figures and Tables

**Table 1:** The result of the fitting analysis.

Sample	*R*_0_/Å	*σ*/Å	*R*_HS_/Å	*ϕ*	*ξ_DB_*/Å
ac-SiO_2_/cPVA/borax	59	0.44	59	0.15	252
ChsNF/ac-SiO_2_/cPVA/borax	59 (fix)	0.44 (fix)	59 (fix)	0.15 (fix)	126

## Data Availability

Data are contained within the article.
